# Crafting safe and effective suicide prevention media messages: outcomes from a workshop in Australia

**DOI:** 10.1186/s13033-018-0203-5

**Published:** 2018-05-24

**Authors:** Maria Ftanou, Jaelea Skehan, Karolina Krysinska, Marc Bryant, Matthew J. Spittal, Jane Pirkis

**Affiliations:** 1 Centre for Mental Health, Melbourne School of Population and Global Health, The University of Melbourne, Carlton, Vic Australia; 2 Everymind, Newcastle, NSW Australia; 3 School of Medicine and Public Health, University of Newcastle, Newcastle, NSW Australia; 4 School of Public Health and Preventive Medicine, Monash University, Melbourne, Australia

## Abstract

**Background:**

Suicide and suicide-related behaviours are major public health concerns in Australia and worldwide. One universal intervention that has received an increased focus as a means of preventing suicide is the use of media campaigns. There is, however, a lack of understanding of the kinds of campaign messages that are safe and effective. The current paper aims to expand on this knowledge. The study objectives were to: (1) explore what suicide prevention experts consider to be essential characteristics of effective and safe suicide media campaigns; (2) develop suicide prevention media messages; and (3) explore the impact that these messages might have on different audiences.

**Methods:**

We conducted a workshop in July 2015 which was attended by 21 experts (professionals with knowledge about suicide prevention and/or media campaigns, and people with a lived experience of suicide). The experts were split into three groups, and each group developed a suicide prevention message for one of the following target audiences: people at risk of suicide; family and peers of people at risk of suicide; and people bereaved by suicide.

**Results:**

The three groups generally agreed that these messages had to include two key characteristics: (1) validate or reflect the target group’s issues and needs; and (2) promote help-seeking behaviours. They noted, however, that messages that might have a positive impact for one target audience might inadvertently have a negative impact for other target audiences. In particular, they were concerned that messages designed for family and peers about being supportive and looking for warning signs might leave those who had been bereaved by suicide feeling isolated, guilty or traumatised. Workshop participants highlighted that gaps exist in relation to the use of appropriate language, were unsure of how to create destigmatising messages without normalising or sensationalising suicide and commented on the lack of evaluative evidence for the efficacy of media campaigns.

**Conclusions:**

Developing suicide prevention messages is complex and target and non-target audiences may interpret these messages differently to the way they were intended and the impact of such messaging may be detrimental. Caution needs to be applied when developing suicide prevention messages.

## Background

Suicide and suicide-related behaviours are major public health concerns in Australia and worldwide. Globally, approximately 800,000 people die by suicide each year [1]. In Australia, a total of 2862 people died by suicide in 2016. This equates to a rate of 11.7 per 100,000 and represents a rise from 10.4 per 100,000 in 2006. Suicide rates are highest in men over 85 years, followed by men in their 30s, 40s and 50s, and suicide continues to be the leading cause of death among people ages 15–44 years [2–4].

The impact of suicide is profound. People who end their own life by suicide often experience significant negative emotions such as shame, guilt, and sadness. Suicide has been closely associated with severe mental health problems [i.e., major depressive disorder, psychosis, post-traumatic stress disorder (PTSD), bipolar disorder, substance abuse and eating disorders], a history of self-harm, a family history of mental illness, and past physical, emotional and/or sexual abuse [5, 6]. More than half of those who die by suicide have previously had contact with mental health services, indicating that people experiencing suicidal thinking endure considerable suffering and distress [1, 7]. Family and friends often worry about people at risk of suicide and often are unsure of how to help.

For every individual who dies by suicide, it has been estimated that an average of 135 people suffer intense grief or are markedly affected [8]. These people can include siblings, parents, grandparents, relatives, friends and the wider community. Those bereaved by suicide have been found to experience high levels of distress, increased feelings of guilt, blame, stigmatization, abandonment and shame. Those bereaved by suicide have also been found to have high rates of psychiatric disorders, suicidal ideation [9], as well as being at a high risk of suicide themselves.

The prevention of suicide is a public health priority [10]. In Australia, a recent National Mental Health Commission review of Australia’s Mental Health System has called for a 50% reduction in suicides and suicide attempts over the next decade [11]. Internationally a multi-level and multi-faceted system approach to combat suicide has been recommended [1, 11]. This approach includes a combination of universal interventions aimed at a whole of population level, selective interventions targeting groups that are identified as high-risk and indicated interventions that specifically target people who are showing signs of suicide risk and suicidal behaviours [1, 11–13].

One universal intervention that has received increasing focus is the use of media campaigns. Campaign messages are often placed on billboards and posters and in pamphlets, and/or are aired on radio, on television, in cinemas or via social media channels. There is a lack of evidence about how messaging in these campaigns should be crafted, and there are concerns that the content of some messages could be counter-productive or potentially do harm [14–17].

We know from elsewhere in the suicide prevention literature that journalists’ reports of a suicide can sometimes have negative consequences, with over 100 studies having explored this relationship. Collectively, these studies have shown that certain types of media reporting of suicide deaths are associated with “copycat acts” or increases in suicidal behaviour. In particular, research indicates that an increased risk of suicidal behaviour has been associated with media messages that have: had repeated coverage; been sensationalised and/or glamorised; explicitly described the method and location of suicide; or portrayed suicide as a likely solution to problems [17–20].

There is, however, a growing body of work that supports the potential positive influence that appropriate messages may have on prevention of suicide [21–26]. Again, some of this evidence comes from studies on media reporting of suicide, rather than on studies of campaigns per se. For example, Niederkrotenthaler et al. [21] found that media reports that focused on adaptive coping or mastery of problems by people facing a suicidal crisis were negatively associated with suicide rates. Other evidence, however, comes more directly from evaluations of campaign messages. For example, Oliver et al. [22] and Jenner et al. [23] observed that simple messages about the preventability of suicide, delivered in the context of a media campaign, led to increased calls to helplines. Others examined the delivery format of media messages and found that that public service announcements (PSAs) were more effective than a billboard simulation in suicide prevention efforts [26].

Understanding of what makes messages in suicide prevention campaigns safe and effective is lacking. The National Institute of Mental Health (NIMH), the Centres for Disease Control and Prevention (CDC), and the Centre for Mental Health Services of the Substance Abuse and Mental Health Services Administration (SAMHSA) convened a 2-day workshop in 2003 on the science of public messages for suicide prevention [27]. This workshop was attended by over 40 experts in the suicide prevention and public health fields. In breakout sessions, workshop participants discussed the impacts of three existing public awareness campaigns. At the end of the workshop, a number of recommendations were made, including that those developing media campaigns: (a) adopt a scientific approach throughout the life of the campaign, from message planning and development to implementation and outcome assessment; (b) pre-test media messages to ensure that any unwanted behaviours are not normalised; (c) consider the impacts of media messages on both the targeted group and non-targeted groups; (d) portray helpful options and solutions; and (e) do not overgeneralise particular risk factors associated with suicidal behaviour (e.g., not over-emphasising the link between depression and suicide, as a viewer may misinterpret the message to mean that suicide is the inevitable result of depression [27].

More than 10 years on from the US workshop, suicide prevention campaigns have become more prominent. We recently conducted an international search for public service announcements that had been screened as part of a small- or large-scale suicide prevention campaign and identified 35, 60% of which were made after 2011 [28]. Despite this, knowledge about the characteristics and content of safe and effective suicide prevention media messages is still lacking. For this reason, we conducted an interactive workshop in 2015 in Australia which was similar to the one held in 2003 in the US, but instead of examining the impacts of pre-existing media campaigns, we explored participants’ views about appropriate content for media messages for three distinct groups: people at risk of suicide; family and peers of people at risk of suicide; and people bereaved by suicide.

This paper reports on the findings from our workshop. Specifically, the study objectives were to: (1) explore what suicide prevention experts consider to be essential characteristics of effective and safe suicide media campaigns; (2) develop suicide prevention media messages; and (3) explore the impact that these messages might have on different audiences.

## Methods

### Interactive workshop

The workshop was held in Hobart, Australia on July 28th 2015 at the National Suicide Prevention conference hosted by Suicide Prevention Australia. This conference is held annually and draws more than 400 suicide prevention researchers, policy makers service providers, advocates, and people with lived experience of suicide, from Australia and overseas.

The workshop was facilitated by JS, KK and JP, with the assistance from MF. The primary goals of the workshop were to:

1. Develop media messages for different target audiences and consider the likely intended and unintended consequences of particular messages; and
2. Identify challenges in creating media messages for suicide prevention.

The workshop began with a short presentation from each of the facilitators. These presentations were designed to orient participants to the task of developing and discussing safe and effective messages for suicide prevention. JP described the findings of our previous study, mentioned above, in which we identified suicide prevention public service announcements from around the world [28]. She noted that the majority of these promoted open discussion about suicide, indicated that the life of a suicidal person was important, acknowledged the suffering associated with suicidal thoughts and feelings, stressed that suicide is preventable and/or promoted support for people at risk of suicide. JS summarised the existing literature on the role of the media in suicide prevention. She highlighted that caution needs to be taken when developing media messages as it is difficult to monitor how information is interpreted by people with varying degrees of risk and that some messages may be misinterpreted and/or have unintended consequences. KK discussed the impact of media messages on people who have been bereaved by suicide, in particular noting that campaign messages such as “suicide is preventable”, “mental health problems are treatable”, and “there are warning signs” may leave bereaved people feeling that they are to blame for the death.

After these presentations, participants were divided into three discussion groups and each group was asked to develop a single media message for one of the three target audiences: (a) people at risk of suicide; (b) people bereaved by suicide; and (c) family and peers of people at risk of suicide. Once they had done this, they were asked to consider the likely consequences of their message for the other two target audiences. Participants were given approximately 40 min to develop and discuss their messages. We did not provide comments during the process, but rather allowed the workshop participants to generate content that was important to them, taking into account their own expertise.

Participants then returned to the larger group, and a spokesperson from each of the three discussion groups relayed their message, described their intent, and summarised the impact they felt they would have on their target audience and on the other two audiences. The full group was then invited to comment, and general discussion ensued about the challenges and gaps in knowledge in relation to creating safe and effective media messages for suicide prevention.

### Data collection and analysis

Key discussion points from each of the breakout sessions were summarised and written up by each group and given to the facilitators. Key points from each spokesperson and responses from the general audience were written on a whiteboard by the facilitators. The workshop was also audio recorded and transcribed for analysis. Workshop data were analysed using a thematic analysis approach. Transcripts were read and re-read for emerging themes and then coded into themes and subthemes.

## Results

### Workshop participants

Twenty-one individuals (12 females and nine males) took part in the workshop. Nine participants (43%) were service providers, six (29%) were researchers, three (14%) were policy-makers, and two (9%) were people with lived experience of being suicidal and/or being bereaved by suicide (membership of multiple categories was permissible). For the breakout sessions, participants were divided into three equal groups of seven to develop messaging for one of the three target audiences and consider the impact of that messaging for the other audiences.

### Target audience (a): People at risk of suicide

#### Development of message

The group that considered messages for people at risk of suicide identified four key characteristics that they thought were important to capture in a suicide prevention media message for people at risk. Firstly, they all agreed that it was crucial to validate and acknowledge the person’s pain and suffering. One participant stressed that without this acknowledgement, people at risk would not connect with the message, saying, *“I think we need to have a reflection. We need to be able to reflect someone’s experience. We need to be able to say, ‘Yeah you feel like there’s no way out’. We have to be able to validate that for people, otherwise they’re going to tune out straight away, and say, ‘You don’t even get it’”.*

Secondly, the group considered it important that their media message highlighted that “the person’s life matters”, that “the person is valued” and that “people care”. One participant, however, expressed concern that people at high risk might not relate to such a positive message about life. This person said, *“You’ve got people on the trajectory that if they’re at a black place completely, telling them messages about ‘life’s good’ is probably not going to be as effective”.*

Thirdly, this group thought it was important to capture the concept of mastery. In particular, they wanted people at risk of suicide to know that although they are suffering, there are alternatives to suicide. Participants in this group also questioned whether media messages about mastering a suicidal crisis would be more effective if delivered by people with lived experience of suicide. The following comments are examples of mastery messages expressed by the group: *“There is a solution, [even though] right now you don’t see it”* and *“There is a way out, and others have [found it], and … come out the other side”.*

Finally, this group wanted to tailor their media message to encourage help-seeking. They wanted to not only stress the availability of services, contact numbers, websites and supports (e.g., *“There are people waiting to talk to you with nothing else to do but wait for your call”*) but also give emphasis to the importance of *“accepting help as a first step”*.

Taking the above four characteristics into account, this group created the message in Box 1 for people at risk of suicide.

#### Box 1: Media message for people at risk of suicide

The pain is deep and it hurts. There seems to be no way out.

You matter. We are here*. Call*

##### Impact of message on non-target audiences

When the group considered the impact of their media message on people bereaved by suicide, the group was concerned that bereaved individuals might not connect with the message. They were concerned that bereaved people might feel that they are not the priority, and that the focus is on suicidal individuals not those who surround them and care for them (e.g., *“The suicide didn’t happen to them”*). They were also worried that the message might result in bereaved people feeling guilty or blaming themselves (e.g., *“[They] failed to tell [their] child that they mattered”*).

When the group explored the impact of their media message for family and peers of people at risk of suicide, the group generally felt that the message was safe but were concerned that it underestimated the role of family and peers and their ability to intervene.

### Target audience (b): People bereaved by suicide

#### Development of message

In developing their media message, the group that was asked to consider people bereaved by suicide discussed the process of grief. In particular, they emphasised that grief is an individual process, characterised by emotional pain, stigma, anger, loneliness, isolation and sometimes shame. They conveyed that a *“large number of people are affected by suicide loss”*, including family, friends, work- or school-mates, and community members. The group identified two main characteristics that they thought were vital to capture in a media message for people bereaved by suicide. Like the group considering messages for people at risk of suicide, this group felt that it was critical that media messages both validated and reflected the bereaved person’s pain and promoted help through professional avenues an informal channels. The following remarks summarises the group discussion: *“We discussed the concept of bereavement. It impacts on everyone. Sadness, isolation … impact on friends, family, community. It’s an individual personal experience… so everybody grieves and we all grieve in our own unique way, but we join together as part of that large group in our grief. And then there’s this general community messaging about you know where to go get help and also come together and help each other … everyone can hear another’s loss.”*

Taking the above two characteristics into account, the group proposed the media message in Box 2 for people bereaved by suicide.

#### Box 2: Media message for people bereaved by suicide

The wound will heal but there will always be a scar. There is no right way to grieve. Everyone can hear another’s loss. There is help, and there are places to share your story. *Call*

##### Impact of message on non-target audiences

When the group reflected on the potential impact of their message for people at risk of suicide, they were concerned that it might have the unintended consequence of making this group feel guilty for having suicidal thoughts, and fear asking for help in order not to burden family and peers. They were also worried that it might lead to this vulnerable group feeling isolated, especially if they perceived themselves lacking emotional and social supports.

For family and peers of people at risk of suicide, the group was concerned that this kind of messaging might lead to family and peers not wanting to reach out to people at risk in case they failed and the consequences were serious.

### Target audience (c): Family and peers of people at risk of suicide

#### Development of message

This group discussed two features that they thought should be portrayed in their media message. Firstly, they felt that it was essential that family and peers be validated regarding the difficulties that they might have in approaching someone at risk of suicide, but at the same time also be given permission *“to ask the question”*. There was debate about whether family and peers should ask generally about well-being or specifically about suicide. One participant said, *“[The message that] it’s okay to ask … [is] … too vague. I would like to know… to ask about what? You know, I think you need to [clearly indicate] suicide. I think the message [that it’s okay to ask about] suicide [needs to be] very clear. It’s got to be direct, honest and open, and I don’t hear that. ‘You’ve got to ask’ is too big for me. So to clarify that, it’s okay to ask about suicide.”*

Secondly, just as the other two groups did, this group wanted their media message to promote the availability of help services, not just for the person at risk of suicide but also for their family and peers. One participant stressed the role of support, saying, *“The message is to let people know that there is support … and that support would be both for the person who is at risk and for the person who is concerned about the person who is at risk. They both need to be able to access support*.”

Consideration of the above two features led the group to propose the message in Box 3.

#### Box 3: Media message for family and peers of people at risk of suicide

It’s okay to ask. Be prepared for the answer. There is support for both of you. Call

##### Impact of message on non-target audiences

In considering the impact of their media message on the other two audiences, these participants were worried that it might contribute to people at risk of suicide feeling like *“passive recipients of help”* and that that it might even encourage *“learned helplessness”*. They were concerned that if someone at risk was exposed to the message and then not asked how they were, this might lead to their feeling isolated and alone which might further impact on their mental state. This concern is exemplified by the following remark: *“Okay, say people weren’t asked, that would just reinforce the fact that they’re completely socially isolated and no one cares, which is potentially really, really bad”.*

The group was concerned that their message might potentially lead to feelings of trauma, guilt and shame in people who had been bereaved by suicide. One participant discussed the thought processes a bereaved person might go through if they were exposed to the message: *“I never actually did ask, so that was blatantly something that you’re telling me that I should’ve done that I didn’t do, it’s quite obvious. Or I didn’t ask in the right way”.*

### Challenges in creating media messages for suicide prevention

The broader discussions held after the groups presented their messages indicated that there are various complexities in developing effective media messages for combating suicide. Three main challenges were identified:

#### General versus specific use of language

Workshop participants were unsure about the use of the word “suicide” in media messages. They were aware of guidelines that discourage prominent use of the word in reports about suicide presented on television or in newspapers but questioned whether the absence of it in the current context might make messages too oblique. Some participants favoured direct use of the word, whereas others were more sanguine about less overt phrases like *“struggling with life”*.

#### Normalising versus destigmatising

Workshop participants thought that media messages should be convincing, relatable and engaging, but were unsure of how to create messages that were destigmatising without simultaneously creating ones that normalised or sensationalised suicide.

#### Whole-of-population versus specific at-risk groups

Workshop participants were unsure whether it would be more beneficial to target the population as a whole or to target specific groups at heightened risk of suicide and those who care about them (e.g., men in rural areas). They were unsure if whole-of-population efforts could meet the needs of the many at-risk groups who make up the population, and whether other suicide prevention activities (e.g., gatekeeper training programs) might be necessary as an adjunct.

The main themes that emerged from the workshop were that the experts considered acknowledging suffering and promoting help seeking to be important features of suicide prevention campaigns. Workshop participants, however, were worried that media messages may have unintended outcomes for non-targeted groups and that destigmatising messages might normalise or sensationalise suicide.

## Discussion

There is a lack of understanding of the characteristics that contribute to safe and effective media campaigns to combat suicide. Ultimately, the aim of media campaigns should be to encourage individuals to take appropriate and helpful actions (e.g., to seek help for suicidal thoughts or to support someone they are concerned about). Our workshop participants suggested that media messages for suicide prevention should include two key characteristics. Firstly, they thought that to positively engage and influence public beliefs and attitudes, the message had to in some way validate or reflect the target audience’s difficulties. Secondly, they believed the message needed to promote help-seeking behaviours and services.

Promoting calling a helpline or other service is a key component of most suicide prevention media messages [28] and leads to people help seeking. For example Oliver et al., in a 2008 campaign adopted the message “Suicide is preventable. Its causes are treatable. For immediate help call (emergency number)”, led to an increase in calls to an emergency mental health service in the US [22]. Similarly, an increase in calls was noted immediately following a suicide prevention billboard campaign stating “There are a lot of reasons to love life. If you are currently unable to find a reason: Call” in Austria [29].

The workshop participants also illustrated the difficulties associated in developing media messages for the prevention of suicide. Despite carefully scripting their media messages and trying to take into account non-target group responses to their media messages, participants were still concerned that people with varying degrees of risk and non-target audiences may interpret the messages differently to the way they were intended, which could lead to some members of the public feeling isolated, guilty or traumatised. These negative effects of suicide prevention messages have been demonstrated in other campaigns. For example, other researchers found that billboard advertisements sometimes led to an increase in maladaptive coping strategies in vulnerable youth [25, 26].

Despite the complexities and lack of evidence, media messaging is being increasingly used as a suicide prevention tool. Suicide preventions messages are being used to promote help services, decrease stigma and increase awareness. For example some Australian organisations have begun to develop small scale suicide prevention campaigns (e.g., Suicide Prevention Australia developed one targeting men; the Australian Medical Association developed another for people affected by natural disasters and other countries have also adopted the campaign approach, often with life-affirming or empathic slogans (e.g., “I am Alive” in the UK; “Choose Life” in Scotland; “We’ve Been There” in the US). There is a critical need for research that evaluates the effectiveness of the content of these media messages [27]. Until the evidence base improves, caution should be used when developing and disseminating media messages for the prevention of suicide.

When planning suicide prevention media messages, focus groups with target and non-target audience members should be utilised to gain an understanding of the kind of information that people with varying degrees of suicidality and/or different relationships to suicidal individuals would like depicted in these messages. The information gleaned from focus group needs to be compared to the latest evidence base in the area and safeguards be put in place to develop messages that are supported by empirical research [27, 30].

To understand how people at risk of suicide feel and what might be helpful for them they need to be consulted and involved in co-designing CSAs with other key stakeholders. In a recent blueVoices study people who had been suicidal were asked  about what people had done or said that was helpful and what people had done and said that was not helpful. Such studies can help work out what the content of a best-practice media campaign might look like [31].

Guidelines on media reporting of suicide such as Australia’s Mindframe guidelines [32] can be used to help shape the development of the message. These guidelines recommend that media reports of suicide should: not portray suicide as a common or acceptable response to adversity; not glamorise, sensationalise or romanticise suicide; not present simplistic explanations for suicide or indicate it is inevitable; not include details such as method, location or personal information; not use stigmatising language; not reinforce myths and stereotypes; and should promote alternatives, positive narratives and help seeking behaviours.

Once the media message has been developed, it is essential that it be pre-tested on both target and non-target audiences before the message is released at a population level [30]. Pre-testing the media message in various at-risk groups (e.g., people with mental illness or drug and alcohol issues, young people, socioeconomically disadvantaged people) may help gain an understanding on the differential impacts the media messages might have.

Pre-testing, however, does not guarantee that the media message will be safe or effective at a population level [30]. Ongoing evaluation of the immediate impacts (e.g., whether the message increases awareness), intermediate impacts (e.g., whether the message increases help seeking behaviours) and long-term impacts (e.g., whether the message reduces suicide attempts and deaths by suicide) should ideally be built into any media message campaign aimed at combating suicide [27].

Our exploration of how suicide prevention messages might be crafted had a number of limitations. Our participants were relatively few in number, were self-selected, and were all English-speaking and predominately female. This raises questions about the extent to which our findings can be generalised. Participants were asked to develop and consider the impact of their messages in a short period of time (i.e., under 2.5 h). These limitations are perhaps offset to a degree by the expert nature of the workshop participants. Participants were up to date with the latest research, were working in the area of suicide prevention and/or had keen interest and/or lived experience. To analyse the data, transcripts were read and re-read for emerging themes and then coded into themes and subthemes. Due to the small sample size, other tools such as spider maps and score cards were not used, which may have enhanced the interpretation of the results [33].

## Conclusions

Developing suicide prevention messages is complex and target and non-target audiences may interpret these messages differently to the way they were intended and the impact of such messaging may be detrimental. Caution needs to be applied when developing suicide prevention messages.
